# Life‐history constraints on maximum population growth for loggerhead turtles in the northwest Atlantic

**DOI:** 10.1002/ece3.5398

**Published:** 2019-08-02

**Authors:** Joshua M. Hatch, Heather L. Haas, Paul M. Richards, Kenneth A. Rose

**Affiliations:** ^1^ NOAA National Marine Fisheries Service, NEFSC Woods Hole MA USA; ^2^ NOAA National Marine Fisheries Service, SEFSC Miami FL USA; ^3^ Horn Point Laboratory University of Maryland Center for Environmental Science Cambridge MD USA

**Keywords:** annual maximum population growth rate, Bayesian, *Caretta caretta*, demography, life‐history parameters, loggerhead turtles

## Abstract

Conservation planning for protected species often relies on estimates of life‐history parameters. A commonly used parameter is the instantaneous maximum population growth rate (*r*_max_) that can be used to limit removals and design recovery targets. Estimation of *r*_max_ can be challenging because of limited availability of species‐ and population‐specific data and life‐history information. We applied a method proposed by Neil and Lebreton, originally developed for birds, to loggerhead turtles. The method uses age‐at‐first‐reproduction and adult survival to estimate *r*_max_. We used a variety of datasets and matrix population models to confirm an allometric assumption required by the method, and to generate estimates of age‐at‐first‐reproduction and adult survival. A meta‐analysis was applied to parameters from reported growth curves, which were then combined with the size distribution of neophyte nesters to derive estimates of age‐at‐first‐reproduction. Adult survival rates were obtained from an existing matrix population model. Monte Carlo simulation was then used to combine the estimates of the allometric coefficients, age‐at‐first‐reproduction, and adult survival to obtain a probability distribution of approximate *r*_max_ values. Estimated annual maximum population growth rates averaged 0.024, with a mode of 0.017 and a 95% highest density interval of 0.006–0.047. These estimates were similar to values reported by others using different methods and captured the variability in positive, annual change estimates across nesting beach sites for the northwest Atlantic loggerhead population. The use of life‐history parameters has a long history in wildlife and fisheries management and conservation planning. Our estimates of *r*_max_, while having some biases and uncertainty, encompassed values presently used in recovery planning for loggerhead turtles and offer additional information for the management of endangered and threatened species.

## 1 INTRODUCTION

The maximum rate of population growth, be it instantaneous (*r*_max_) or finite ($\lambda_{\max}$), is an important demographic parameter for the conservation and management of marine wildlife (e.g., marine mammals, sea turtles, sharks, and seabirds). The maximum population growth rate is achieved under conditions of high resources and availability of habitat, and often when the population is at low abundance so that density‐dependent survival and reproduction are minimal. In age‐ or size‐structured populations, maximum population growth is realized when the population is at or near its stable age/size distribution.

**Figure 1:**
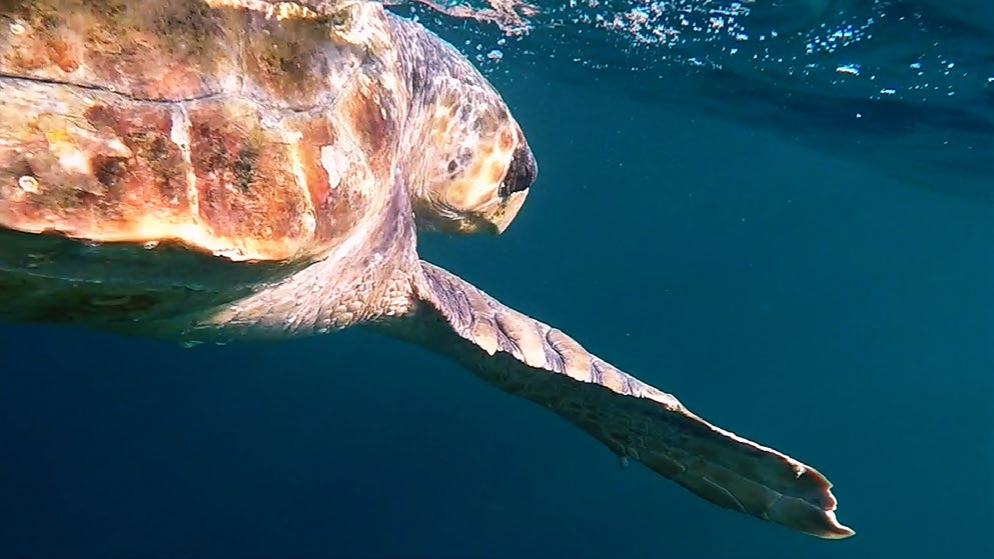
Photograph of a loggerhead sea turtle (*Caretta caretta*) taken in collaboration with NOAA, NMFS, NEFSC and the Coonamessett Farm Foundation under Endangered Species Act Permit No. 18526

In conservation, the maximum rate of population growth can be used to define management objectives that include setting recovery criteria or limiting removals from threatened populations (Curtis & Moore, 2013; NMFS and USFWS, 2008; Wade, 1998). A general approach for setting conditions on removals from threatened populations uses limit reference points (LRPs; Curtis, Moore, & Benson, 2015), such that removal estimates that exceed an LRP trigger subsequent management action. Examples of LRPs for protected species include the Potential Biological Removal (PBR, Wade, 1998) and the Reproductive Value Loss Limit (RVLL, Curtis & Moore, 2013); both are functions of the maximum population growth rate. Other assessment approaches, often used for loggerhead sea turtles (*Caretta caretta*; see Figure 1), that could benefit from estimates of maximum population growth include qualitative evaluations of jeopardy (U.S. Dept. of Commerce 2001) and population viability analyses (Merrick & Haas, 2008; Snover & Heppell, 2009).

For many long‐lived and late‐maturing species, the maximum rate of population growth is unknown or inestimable using direct methods (e.g., analysis of trends in abundance indices) because of data limitations (Dillingham et al., 2016). In such cases, demographic models, such as life tables and projection matrices, are used to represent population dynamics and to generate estimates of population growth (Caswell, 2001; Zerbini, Clapham, & Wade, 2010). Because our interest is estimation of the maximum population growth rate, the demographic parameters (e.g., survival, growth, and fecundity) used in these models must be appropriate to optimal conditions for population growth (i.e., abundant resources and habitat and minimal human impacts). Measuring optimal demographic rates is challenging, and estimates (either derived from data or from models) typically have considerable uncertainty. This high uncertainty has led researchers to rely on filters that exclude unrealistic population growth rates (i.e., too low or too high) from the estimation (SEFSC 2001, Warden, Haas, Richards, Rose, & Hatch, 2017), which hinders understanding of how these demographic parameters that underlie population growth can feasibly combine to constrain and allow estimation of an optimal population growth rate.

Niel and Lebreton (2005) developed a method to estimate the maximum rate of population growth in data‐limited situations that requires only estimates of adult survival and age‐at‐first‐reproduction. Initially applied to birds (Dillingham & Fletcher, 2008), this method was extended to a variety of taxa that included mammals and sharks (Dillingham et al., 2016). When compared across shark populations with various combinations of values for life‐history parameters, Neil and Lebreton's method generated similar (albeit somewhat lower) estimates of maximum population growth rates as other commonly used data‐limited approaches (Cortés, 2016). The limited data requirements of Neil and Lebreton's method, combined with indications from comparative studies like Cortés (2016), make it a viable approach for estimating maximum population growth rates from life‐history parameters in knowledge‐sparse situations.

In this paper, we develop a plausible distribution of maximum population growth rates for the northwest Atlantic (NWA) population of loggerhead turtles using the method outlined in Niel and Lebreton (2005). The northwest population is also listed as the northwest Atlantic Distinct Population Segment under the U.S. Endangered Species Act (ESA). We incorporate uncertainty in adult survival and age‐at‐first‐reproduction by means of a Monte Carlo approach and follow Dillingham et al. (2016) by allowing the derived allometric constant to vary, thereby allowing us to generalize our findings to the population of northwest Atlantic loggerhead turtles. Probability distributions around key parameters were either inferred from prior literature or were developed through meta‐analytics. We discuss the strengths and limitations of our results in the context of using life‐history parameters, and specifically maximum population growth rates, for management of protected species.

## 2 THEORY: ESTIMATING *r*_max_ FROM LIFE‐HISTORY PARAMETERS

Neil and Lebreton's method is based on demographic invariant theory and relies on certain allometric relationships,

(1) $$r_{\max}\approx a_{r}M^{-0.25}$$

and

(2) $$T_{\mathrm{op}}\approx a_{T}M^{0.25}$$

where M is body mass and $T_{\mathrm{op}}$ is the optimal generation time. Combining the two equations results in,

(3) $$r_{\max}T_{\mathrm{op}}\approx a_{r}a_{T}M^{0}=a_{r}a_{T}$$

where $a_{r}a_{T}$ is a dimensionless, allometric constant (Niel & Lebreton, 2005). Considering discrete exponential growth, the maximum fractional amount a population could grow in one generation is $\lambda_{\max}^{T_{\mathrm{op}}}$. On the log scale, this becomes $T_{\mathrm{op}}\ln(\lambda_{\max})$, which, in terms of the instantaneous growth rate, is equivalent to $r_{\max}T_{\mathrm{op}}$. Given an age‐at‐first‐reproduction (α), and assuming constant adult survival (s) and fecundity after the onset of reproduction, the optimal generation time reduces to,

(4) $$T_{\mathrm{op}}=\alpha +\frac{s}{\lambda_{\max}-s}$$

(Niel & Lebreton, 2005). Combining equations 3 and 4 yields,

(5) $$\lambda_{\max}=\exp\left[a_{r}a_{T}{\left(\alpha +\frac{s}{\lambda_{\max}-s}\right)}^{-1}\right].$$

In order for Equation (5) to be solvable, a value for $a_{r}a_{T}$ must be assumed or estimated, with Dillingham et al. (2016) showing that $a_{r}a_{T}\approx 1$ for a variety of taxa (i.e., mammals, birds, and sharks). Dillingham et al. (2016) also recommended further research into the value of $a_{r}a_{T}$ for other taxa, with turtles being of particular interest. A key difference in Equation (5), relative to matrix population models (MPMs), is that underestimation of adult survival will inflate $\lambda_{\max}$.

## 3 METHODS: APPLICATION TO LOGGERHEAD TURTLES

**Figure 2:**
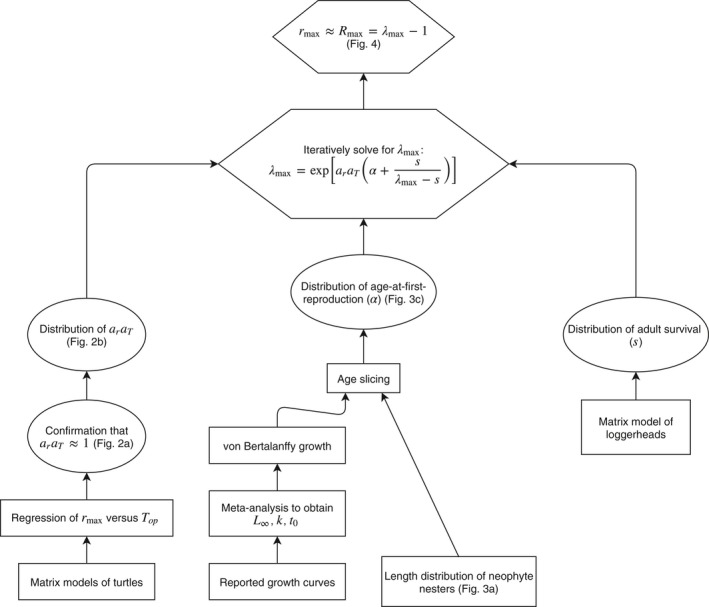
Flowchart of the method used to estimate the approximate maximum population growth rate, *R*_max_, for the Northwest Atlantic population of loggerhead turtles. $\lambda_{\max}$ is the finite maximum population growth rate, $a_{r}a_{T}$ is the allometric constant, and $L_{\infty }$, k, and $t_{0}$ are parameters of the von Bertalanffy growth curve

The series of calculations and data sources used to apply the Niel and Lebreton (2005) method to loggerhead turtles is shown schematically in Figure 2. We first confirm the assumption of the allometric relationship ($a_{r}a_{T}\approx 1$, leftmost pathway in Figure 2) using regression analysis of optimal generation time and *r*_max_ based on reported matrix population models for turtles. Second, we use parameters from reported growth curves and the size distribution of neophyte nesters to derive estimates of age‐at‐first‐reproduction (two middle pathways in Figure 2). Third, we use adult survival rates from an existing matrix model of loggerhead turtles (rightmost pathway in Figure 2) that explored sensitivity of population growth to alternative estimates of survival rates (Warden et al., 2017). The adult survival rates, combined with age‐at‐first‐reproduction and assumed values for the allometric constant, enable the estimation of the finite maximum population growth rate ($\lambda_{\max}$), which then enables an approximation (*R*_max_) of the instantaneous version (*r*_max_). Hereafter, *R*_max_ (the finite approximation of the instantaneous maximum population growth rate) will be referred to as the approximate maximum population growth rate. All calculations were implemented in the R statistical computing environment (R Core Team 2018), with estimation using the rjags library (Plummer, 2018) and the Markov Chain Monte Carlo (MCMC) algorithms of Just Another Gibbs Sampler (Plummer, 2017).

The benefit of using Equation (5) to derive $\lambda_{\max}$ is that it only requires estimates of age‐at‐first‐reproduction and adult survival, assuming the dimensionless allometric constant ($a_{r}a_{T}$) is known. Assuming constant adult survival appears reasonable for the NWA population of loggerhead turtles (Warden, Haas, Rose, & Richards, 2015; Warden et al., 2017), as survival senescence is thought to be negligible for most turtle species (i.e., negligible declines in adult survival with increasing age; Wallace, Heppell, Lewison, Kelez, & Crowder, 2008). Conversely, using an MPM to derive estimates of $\lambda_{\max}$ would require values for various demographic parameters under optimal conditions conducive to maximum population growth. For example, a recent MPM for the northwest Atlantic loggerhead population used quantitative information on stage durations, stage survival rates, nests per female, eggs per nest, egg survival, proportion female, and remigration intervals (Warden et al., 2015). By making some simplifications, we can reduce the number of demographic parameters needed to approximate $r_{\max}$; although, we should continue to verify the assumptions of that simplification for turtles.

### 3.1 The allometric constant for turtles

**Figure 3:**
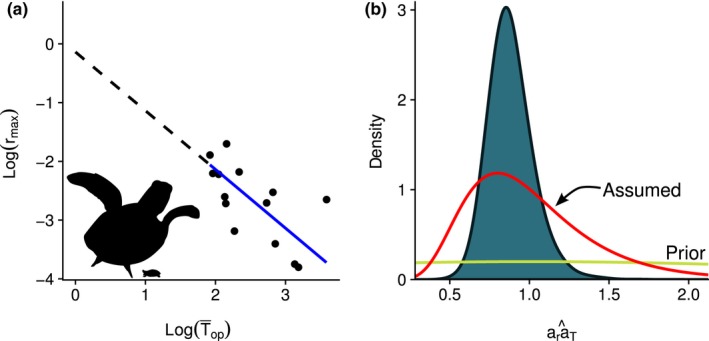
(a) Log‐log regression of optimal generation time (${\bar{T}}_{\mathrm{op}}$) and instantaneous maximum population growth rate ($r_{\max}$) fixing the slope at −1, as predicted by Equation (6). The relative size range of considered turtles is shown in the lower, left‐hand corner. (b) Marginal posterior distribution of $\hat{a_{r}a_{T}}$ from the Bayesian regression estimating only the intercept (filled density curve). Superimposed on (b) is the prior distribution for $a_{r}a_{T}$ used in the Bayesian regression and the assumed distribution for $a_{r}a_{T}$ used in the Monte Carlo estimates of $\lambda_{\max}$ for the northwest Atlantic loggerhead population

The COMADRE database, an open‐online repository for MPMs, was searched for entries related to turtles (Order: *Testudines*; Salguero‐Gómez et al., 2016). Only those entries that had a finite population growth rate (λ) ≥1 were kept for further analysis, as we were interested in population dynamics under demographically optimal conditions (Table 1). Where possible, we confirmed the MPMs reported in the database using corresponding reference documents and made corrections if appropriate. If provided, results from the “best case” scenario of the MPMs were used or we used values that maximized the population growth rate using reported probability distributions around the parameters. We then extracted these “best case” or “maximized” values of $\lambda_{\max}$ and ${\bar{T}}_{\mathrm{op}}$ from each of the selected MPMs (*n* = 14). Here, we used the mean optimal generation time calculated as ${\bar{T}}_{\mathrm{op}}=\frac{\lambda_{\max}v^{⊤}w}{v^{⊤}Fw}$ (Bienvenu & Legendre, 2015), where ⊤ is the transpose operator, F is the fertility matrix, v is the vector of reproductive values, and w is the stable age or stage distribution (see Figure 3a).

**Table 1:** List of population matrix models (MPMs) for turtles extracted from COMADRE, except for Heppell, Crowder, and Crouse (1996)

Species	Reference	Type
Broad‐shelled turtle	Spencer and Thompson (2005)	Stage
Common mud turtle	Frazer, Gibbons, and Greene (1991)	Age
Common musk turtle	Mitchell (1988)	Hybrid
Common snapping turtle	Salice, Rowe, and Eisenreich (2014)	Hybrid
	Zimmer‐Shaffer, Briggler, and Millspaugh (2014)	Stage
Diamondback terrapin	Mitro (2003)	Hybrid
	Crawford, Maerz, Nibbelink, Buhlmann, and Norton (2014)	Stage
Kemp's ridley sea turtle	Heppell et al. (1996)	Stage
Loggerhead sea turtle	Warden et al. (2015)	
Macquarie turtle	Spencer and Thompson (2005)	Stage
Painted turtle	Tinkle, Congdon, and Rosen (1981)	Age
	Mitchell (1988)	Hybrid
Smooth, spiny softshelled turtles	Zimmer‐Shaffer et al. (2014)	Stage
Spotted turtle	Enneson and Litzgus (2008)	Stage

Following Niel and Lebreton (2005) and Dillingham et al. (2016), we used log‐log regression to test underlying assumptions on the relationship between *r*_max_ and ${\bar{T}}_{\mathrm{op}}$,

(6) $$\begin{array}{cc} r_{\max}{\bar{T}}_{\mathrm{op}}\; & \approx a_{r}a_{T}\\ \ln\left(r_{\max}\right)+\ln\left({\bar{T}}_{\mathrm{op}}\right)\; & \approx \ln(a_{r}a_{T})\\ \ln(r_{\max})\; & \approx -\ln\left({\bar{T}}_{\mathrm{op}}\right)+\ln\left(a_{r}a_{T}\right) \end{array}$$

Put in a linear regression context,

(7) $$\ln\left(r_{\max}\right)=\beta_{1}\ln\left({\bar{T}}_{\mathrm{op}}\right)+\beta_{0}+\varepsilon$$

where $\beta_{1}$ is the slope, $\beta_{0}=\ln\left(a_{r}a_{T}\right)$ is the intercept, and $\varepsilon ∼\text{Normal}(0,\sigma^{2})$. We used a Bayesian estimator to solve Equation (7), with vague priors placed on $a_{r}a_{T}∼\text{Normal}\left(\mu =1,\sigma =2\right)T\left(0,\right)$ (where *T* = truncated; i.e., truncated normal) and σ∼Uniform(0,1). The prior distribution of $a_{r}a_{T}$ appears to be flat in Figure 3b because of the high variance assumed for $a_{r}a_{T}$, and using a uniform prior distribution for σ is common practice. The slope, $\beta_{1}$, was fixed at −1 (as predicted by Equation (6)) after it was determined that when $\beta_{1}$ was allowed to vary and be estimated as part of the regression, the estimated value did not significantly differ from −1. Given the low sample size (*n* = 14) and limited contrast in life‐history estimates for the turtle species we examined (i.e., relatively similar values of $r_{\max}$ and ${\bar{T}}_{\mathrm{op}}$), fixing the slope at −1 was considered appropriate and similar to what was done by Niel and Lebreton (2005) and Dillingham et al. (2016). The estimate of the slope represents the value of $a_{r}a_{T}$ for an archetypal turtle population (i.e., mean), with variation in the estimate of $a_{r}a_{T}$ (see Figure 3b) reflecting differences in life history across the examined turtle species and populations. However, we would not expect the distribution for the mean value of $a_{r}a_{T}$ to accurately reflect the variability at the level of an individual population. Therefore, we assumed a wider distribution for $a_{r}a_{T}$ when estimating *R*_max_ for the NWA loggerhead population (see the “Assumed” distribution in Figure 3b).

### 3.2 Age‐at‐first‐reproduction

**Figure 4:**
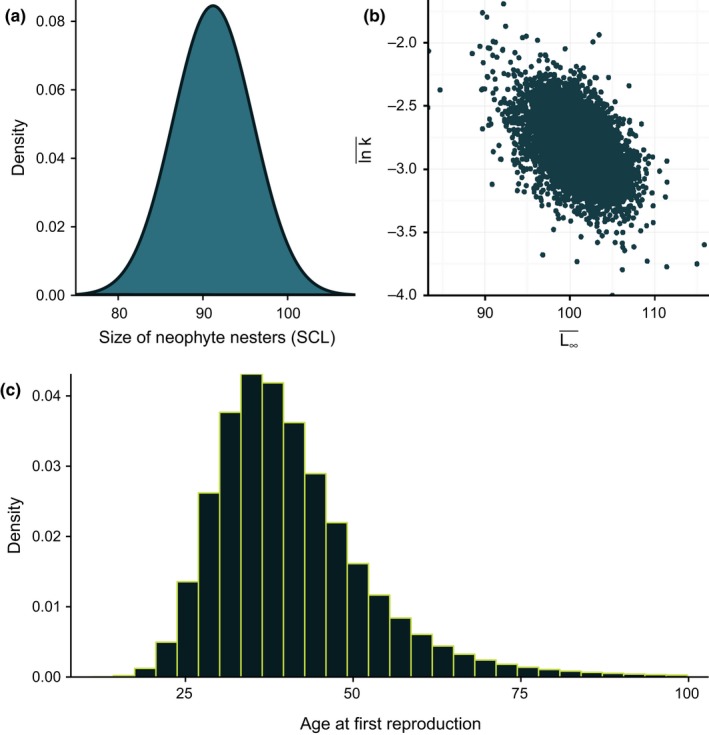
(a) Size distribution of putative, neophyte nesters from the TEWG (2009), (b) joint posterior distribution of $\bar{L_{\infty }}$ and $\bar{\ln k}$ from the meta‐analysis of von Bertalanffy growth curves using mark‐recapture data, and (c) distribution of age‐at‐first‐reproduction developed from (a) and (b) for the northwest Atlantic loggerhead population

A probability distribution of estimated values of age‐at‐first‐reproduction (α) was developed by converting the size distribution of putative, neophyte nesters from the TEWG (2009) (see Figure 4a) using an age slicing technique. Age slicing uses observed lengths and translates them into age using the inverse of a parameterized growth curve. A variety of methods exist to explore size‐at‐age data (e.g., mark‐recapture, skeletochronology, length frequency analysis) for the northwest Atlantic population of loggerheads, and the predominate approach makes use of mark‐recapture data to fit von Bertalanffy growth curves (see citations provided in Table 2). Meta‐analysis provides one way to summarize estimates from these previous studies to gain insight into the individual growth dynamics of loggerhead turtles in the northwest Atlantic. Bayesian hierarchical methods are commonly used in meta‐analyses (Helser & Lai, 2004; Pilling, Kirkwood, & Walker, 2002) and offer a way to explore variability in growth across multiple studies that have spanned varying periods of time and different geographical areas.

**Table 2:** Published parameters (L_∞_ and *k*) from von Bertalanffy growth curves estimated from tag‐recapture data for northwest Atlantic loggerheads, taken mostly from SEFSC (2001) and Vaughan (2009)

Reference	L_∞_ (cm SCL)	*k* (/year)
Avens et al. (2015)^a^	112.35	0.0440
Braun‐McNeill, Epperly, Avens, Snover, and Taylor (2008)	106.90	0.0521
Foster (1994)	96.74	0.0637
Frazer (1987)	94.70	0.1150
Henwood (1987)	110.00	0.0313
Schmid (1995)^b^	96.08	0.0586
Schmid (1995)^c^	96.10	0.0573
SEFSC (2001)	99.70	0.0530
Vaughan (2009)	102.98	0.0759

a Values averaged across estimates for females and males.

b Compiled from all data in the study.

c Compiled from occasions where the intervals between capture and recapture was ≥1 year.

An advantage of using the von Bertalanffy growth equation is that its inverse is easily derived and can be used to obtain age from length frequency information. von Bertalanffy growth is described by,

(8) $$L_{t}=L_{\infty }\left(1-e^{-k(t-t_{0})}\right)$$

where $L_{\infty }$ is the asymptotic length, k is the Brody growth coefficient, and $t_{0}$ is the hypothetical age of length‐0 individuals. By re‐arranging Equation (8), we find that,

(9) $$t=\frac{\ln\left(1-\frac{L_{t}}{L_{\infty }}\right)}{-k}+t_{0}$$

In the fisheries literature, this method of translating length into age is known as age slicing and has several known biases (Sparre & Venema, 1992). First, all individuals greater than $L_{\infty }$ must be removed from the analysis or be arbitrarily assigned to older age classes by the researcher. Second, Equation (9) will assign unrealistically large ages to individuals that are approaching $L_{\infty }$. Despite these flaws, age slicing is one approach that allows prior information on loggerhead turtle growth to be used as a means to approximate the probability distribution of age‐at‐first‐reproduction. For loggerhead turtles, we assumed a maximum age of 100 (i.e., $t_{\max}=100\approx 3\;\text{generations}$) and removed age estimates that exceeded $t_{\max}$ from the analysis.

Previous studies primarily used Fabens’ (1965) method to estimate von Bertalanffy growth parameters from mark‐recapture data (see Table 2). In this case, $t_{0}$ cannot be estimated (and is subsequently not reported) and must be approximated by re‐arranging the von Bertalanffy growth function,

(10) $$t_{0}=t+\left(\frac{1}{k}\right)\ln\left(\frac{L_{\infty }-L_{t}}{L_{\infty }}\right).$$

Assuming the hatchling size for loggerheads in the U.S. northwest Atlantic is ~4.5 cm (LeBlanc et al., 2014) and setting t=0, $t_{0}$ can be calculated using Equation (10) (Natanson, Casey, Kohler, & Colket, 1999).

If we then assume that the parameter estimates (θ) from all prior studies were drawn from a common distribution, then this will lay the conceptual groundwork for a meta‐analytic approach. In other words, we assume that $L_{\infty }$ and k from each published study i were random samples from a bivariate normal‐lognormal (NLN) distribution,

$$\begin{array}{cc} \theta_{i} & ∼\mathrm{BVN}\left(\mu ,\sum \right)\\ (\begin{array}{c} L_{\infty ,i}\\ \ln k_{i} \end{array}) & ∼\mathrm{BVN}(\begin{array}{c} \left[\begin{array}{c} \bar{L_{\infty }}\\ \bar{\ln k} \end{array}\right],\left[\begin{array}{cc} \sigma_{L_{\infty }}^{2} & \sigma_{L_{\infty },\ln k}\\ \sigma_{\ln k,L_{\infty }} & \sigma_{\ln k}^{2} \end{array}\right] \end{array}) \end{array}$$

Here, we model lnk instead of k to ensure that the Brody growth coefficient remains positive on the raw scale during estimation (Helser & Lai, 2004; Pilling et al., 2002). For computational convenience, it is easier to parameterize the NLN distribution in terms of its precision matrix (i.e., $\Omega =\sum^{-1}$). This allows the Wishart (W) distribution to be used as a prior for the precision matrix,

$$\Omega ∼W\left(R=I,k=2\right)$$

where ***R*** is a positive definite m×m matrix (i.e., 2 × 2), which was set equal to the identity matrix (***I***), and k≥m is the degrees of freedom (Plummer, 2017). Priors on **μ** were univariate normal distributions with fixed precisions small enough to be considered vague (i.e., 1E−3). In essence, we are trying to develop an objective method to capture the trajectory of length with increasing age over the lifetime of a loggerhead turtle, with associated uncertainty, given the information contained in previous studies. The marginal posterior distributions of $\bar{L_{\infty }}$ and $\bar{\ln k}$ were then used to construct the distribution of age‐at‐first‐reproduction (see Figure 4b).

While the interpretation of $L_{\infty }$ and k changes depending upon whether length‐at‐age or mark‐recapture data were used in fitting the von Bertalanffy growth equation (Francis, 1988), from a practical perspective, combining estimates based on both types of data has shown promise for long‐lived fishes (Hamel et al., 2014). Furthermore, our goal was not to accurately estimate age from length, but rather to characterize uncertainty in α using a size distribution of putative, neophyte nesters obtained from nesting beach surveys (TEWG, 2009).

### 3.3 Adult survival

The estimates for adult survival (s) were taken from Warden et al. (2015), who adjusted previously reported values of adult survival for bycatch (anthropogenic‐related mortality that is incidental to commercial fishing). Ideally, we would want to use adult survival estimates that reflected conditions in the absence of anthropogenic‐related mortality. Warden et al. (2015) adjusted estimated adult survival rates for bycatch, which is thought to be the biggest source of anthropogenic‐related mortality for the NWA loggerhead population (TEWG, 2009). However, this adjustment does not account for other sources of anthropogenic‐related mortality (e.g., vessel strikes, interactions with recreational fisheries) that are less easily quantified. Following Warden et al. (2017), adult survival was assumed to follow a truncated Beta distribution with a mean of 0.841 and a standard deviation of 0.035 and with lower and upper bounds of 0.770 and 0.925.

### 3.4 *R*_max_

The distribution of *r*_max_ was approximated by $R_{\max}=\lambda_{\max}-1$ (Niel & Lebreton, 2005), where $r_{\max}=\ln\left(\lambda_{\max}\right)$. We decided to use an approximation of the instantaneous maximum population growth rate, *R*_max_, because it provides a slightly more optimistic estimate. $\lambda_{\max}$ was estimated iteratively by Equation (5), drawing values for the allometric constant ($a_{r}a_{T}$), age‐at‐first‐reproduction (α), and adult survival (s) from our specified or developed probability distributions (*n* = 10^6^). For the allometric constant, we drew values from a log‐normal distribution with a mean of 1 and a standard deviation of 0.4 (see Dillingham et al., 2016; Figure 3b). We used the developed distribution shown in Figure 4c for the age‐at‐first‐reproduction values (see Age‐at‐first‐reproduction in the Section 3).

## 4 RESULTS

### 4.1 The allometric constant for turtles

The slope of optimal generation time to instantaneous maximum population growth rate (Figure 3a) did not significantly differ from −1, with the 95% highest density interval for ${\hat{\beta }}_{1}$ ranging from −1.40 to −0.16. We note that the uncertainty in the slope estimate may be underestimated if there is strong phylogenetic dependence in the demographic rates of the turtle species examined. Upon fixing the slope at −1, the marginal posterior distribution for $a_{r}a_{T}$ was estimated and is shown in Figure 3b. We conclude that $a_{r}a_{T}\approx 1$ (mean = 0.89, 95% highest density interval of 0.64–1.17) for turtles, as has been found for birds, mammals, and sharks (Dillingham et al., 2016). This implies that, like other taxa, archetypical turtle populations can no more than triple their abundance in a single generation (i.e., $\lambda_{\max}^{T_{\mathrm{op}}}\approx \exp(1)\approx 3$).

### 4.2 Age‐at‐first‐reproduction

The size distribution of putative, neophyte nesters (Figure 4a) was normally defined with a mean of 91.2 cm Standard Carapace Length (SCL) and a standard deviation of 4.72 cm SCL (TEWG, 2009). The joint posterior distribution of $\bar{L_{\infty }}$ and $\bar{\ln k}$ (Figure 4b) had posterior means of 101.14 cm SCL and 0.06 per year, respectively. Finally, the developed distribution of age‐at‐first‐reproduction (Figure 4c) had modal, median, and mean values of 35, 39, and 41 years; with a 95% highest density interval of 20–63. These findings agree with other published studies (Table 2 of Avens et al., 2015), which reported a range of age‐at‐sexual‐maturity between 14 and 50 years for the northwest Atlantic loggerhead population.

### 4.3 *R*_max_

**Figure 5:**
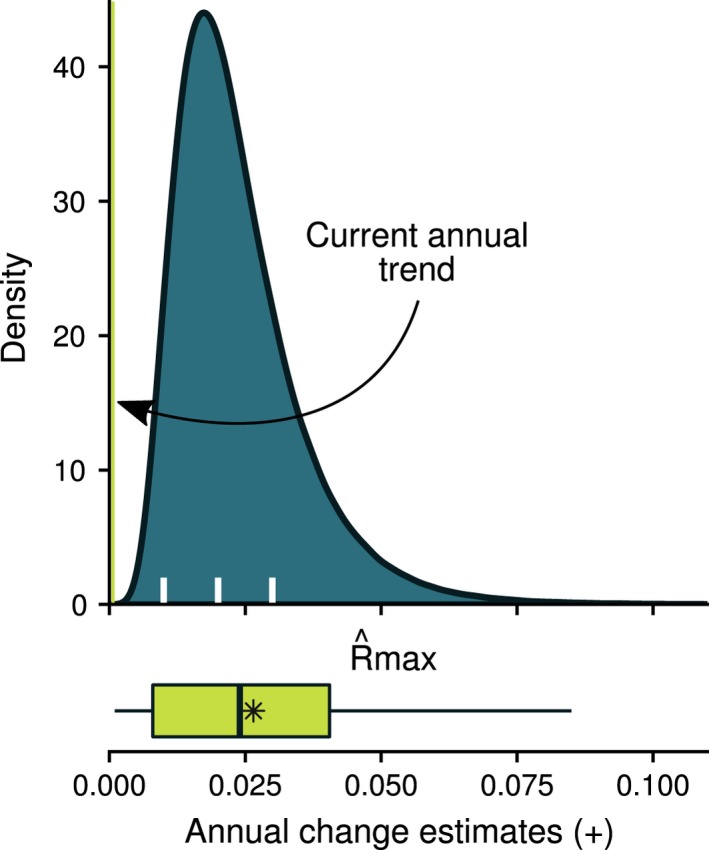
Estimated distribution of the approximate maximum population growth rate for the northwest Atlantic loggerhead population (filled density curve; *R*_max_), along with the positive, annual change estimates from nesting beach sites (boxplot; asterisk denotes the mean; outlier not shown) as reported by Ceriani and Meylan (2017). The distribution of ${\hat{R}}_{\max}$ was developed using the demographic invariant method of Niel and Lebreton (2005) and a Monte Carlo approach. The nesting beach site data included information from 86 nesting beach sites across the U.S. and Mexico spanning all recovery units except the Dry Tortugas, of which 60 sites showed positive trends (Ceriani & Meylan, 2017). The line denotes the current, annual population trend of ~0.0007 as reported in Ceriani and Meylan (2017). White tick marks denote the target demographic recovery rates from the northwest Atlantic loggerhead recovery plan (NMFS and USFWS 2008)

The estimated distribution of the approximate maximum population growth rate (*R*_max_) encompassed the range of published positive, annual growth rates for the northwest Atlantic loggerhead population (Figure 5). The modal, median, and mean values of ${\hat{R}}_{\max}$ were 0.017, 0.021, and 0.024; with a 95% highest density interval of 0.006–0.047. This is in contrast to the current, annual population trend of ~0.0007 reported by Ceriani and Meylan (2017). The estimated *R*_max_ distribution captured the variability in positive, annual growth rates observed across nesting beach sites for the northwest Atlantic loggerhead population (Figure 5). We caution that annual growth rates measured at individual nesting beaches should not be used as an estimate of the approximate maximum population growth rate. We use this comparison to show, at least qualitatively, that our developed distribution of *R*_max_ incorporates the observed intraspecific variation in local population growth rates of the northwest Atlantic loggerhead turtle.

## 5 DISCUSSION

The use of life‐history parameters to inform management decisions has a long history in the fisheries and wildlife sciences. Life‐history parameters have been used to set sustainable harvest rates and yield, to determine the effects of selectivity patterns and fishing mortality on harvest, and to obtain management reference points and subsequent stock status (Beddington & Kirkwood, 2005; Carruthers et al., 2014). However, several life‐history parameters can be difficult to estimate for specific populations (e.g., natural mortality, steepness). The steepness parameter determines the shape of the stock‐recruitment relationship and is defined as the fraction of recruitment from an unfished population (maximum recruitment) obtained when the spawning stock is at 20% of the unfished level (Sharma, Porch, Babcock, Maunder, & Punt, 2019). Natural mortality and steepness have a profound effect on stock assessment output and the resulting management advice (Sharma et al., 2019). Even species with extensive fishery‐independent surveys may be data limited with respect to certain important life‐history parameters. To overcome these limitations, researchers rely on relationships among parameters, often making use of phylogenetic dependencies and meta‐analytics, to inform estimates of poorly defined or unknown life‐history parameters (Thorson, Munch, Cope, & Gao, 2017).

The conservation and management of protected species (i.e., threatened or endangered) is often done in the context of limited data, particularly with respect to life‐history parameters. Limit reference point approaches to bycatch management for sea turtles, while conceptually straight forward, require estimates of life‐history parameters that have a reasonable level of accuracy and precision, which is unlikely in most data‐limited circumstances. Life‐history parameters may also be used to define recovery criteria for the downlisting or delisting of endangered or threatened species (NMFS and USFWS 2008).

The maximum rate of population growth is an important life‐history parameter for marine wildlife, with management sometimes relying on default values defined at higher taxonomic levels (e.g., pinnipeds and cetaceans; Dillingham et al., 2016). Refining and characterizing the uncertainty in life‐history parameters is important to give managers the information needed to navigate difficult decisions in a transparent fashion. Here, we present a frame of reference for the northwest Atlantic loggerhead population's maximum growth rate, *R*_max_, that makes use of a diversity of available information and may be, along with other information, useful for assessing the status of the population.

Recovery plans for endangered or threatened species under the U.S. ESA often identify criteria by which to measure progress toward achieving recovery. A key recovery criterion in the northwest Atlantic loggerhead recovery plan is ensuring positive, annual population growth rates, as inferred by increasing numbers of nests and nesting females (NMFS and USFWS, 2008). The Northwest Atlantic Loggerhead Recovery Team partitioned the northwest Atlantic loggerhead population into five recovery units. Each recovery unit, then, is required to demonstrate a 1%–3% annual increase in the nest and nesting female abundance over a 50‐year time period (i.e., generation time). The exact percentage increase used to define recovery, at least in demographic terms, varies depending upon the recovery unit in question.

In general, we would expect realized, annual instantaneous population growth rates to be lower than *R*_max_. However, spatially varying ecological pressures can complicate the situation, particularly when recovery units of a population are considered separately, as in the northwest Atlantic loggerhead recovery plan (NMFS and USFWS, 2008). For example, a recovery unit could have a high realized, annual growth rate if it sustained immigration from other recovery units or if there are favorable local conditions that are not reflected in the life‐history parameters used to estimate *R*_max_. Because turtles from different recovery units mix on the foraging grounds and because there is no strong evidence that recovery units have different maximum population growth rates, we used available information to create a single distribution of *R*_max_ across recovery units. If recovery units are all part of the same DPS and experience similar conditions in terms of life history (in the absence of human impact), then it may be reasonable to assume that all recovery units would have similar values for *R*_max_, albeit with some natural, random variation.

If we assume that the northwest Atlantic population of loggerheads is at or near a stable age distribution, then we can compare nest count trends to estimated values of *R*_max_. Some of the annual nest count trend targets in the northwest Atlantic loggerhead recovery plan (NMFS and USFWS 2008) are close to or exceed our estimated median annual maximum population growth rate of 2.1% (Figure 4). In particular, the target for the Northern Recovery Unit requires a 2% annual increase, and the targets for the Dry Tortugas and Northern Gulf of Mexico Recovery Units require a 3% annual increase in the nests and nesting females over 50 years. If the estimated distribution of approximate maximum population growth rates presented in Figure 5 is sufficiently accurate, then these demographic recovery targets seem relatively optimistic. The Peninsular Florida Recovery Unit, which may constitute >80% of nesting female abundance (TEWG, 2009), requires a slightly lower demographic recovery target of 1% increase annually that is well within our estimated values. Because *R*_max_ represents the instantaneous maximum population growth rate under optimal conditions, our estimated distribution provides an additional context with which to view the target demographic recovery criteria for the northwest Atlantic loggerhead population.

Best practices for improved recovery criteria under the U.S. ESA include providing demographic criteria that are quantitative and biologically justified (Doak et al., 2015). The northwest Atlantic loggerhead recovery plan provides direct, quantifiable measures of demographic recovery in terms of each recovery unit's risk of extinction. While there is a lot of variability in the annual trends across nesting beach sites (see Figure 5), our estimated distribution of *R*_max_ provides additional information on biologically justified maximum growth rates for the northwest Atlantic loggerhead population. Consideration of such additional information may be useful when developing future demographic recovery criteria.

Our estimated distribution of *R*_max_ (Figure 5) encompasses the upper bounds of the annual population growth rates from demographic models that considered ranges of parameters (2%; Crouse, Crowder, & Caswell, 1987, 6%; Crowder, Crouse, Heppell, & Martin, 1994, 2%; Heppell, Crowder, Crouse, Epperly, & Frazer, 2003). Although Crowder et al. (1994) estimated a possible 6% annual population growth rate, they also discuss a rate of 3.6%/year as being reasonable given simulated mortality reductions. While a direct comparison is not appropriate because none of these studies were estimating *R*_max_, they collectively show a range of plausible values for the upper bound of annual population growth. The cited range of plausible values from 2% to 6% were produced from MPMs (Crouse et al., 1987; Crowder et al., 1994; Heppell et al., 2003) and align well with the estimated distribution of *R*_max_ (see Figure 5), with MPMs often producing less precise values for $\lambda_{\max}$ relative to the method of Niel and Lebreton (2005) (Dillingham et al., 2016). In addition, a status review of loggerheads (Conant et al., 2009) performed a sensitivity analysis of the demographic matrix they used for a threat assessment and found that even with juvenile and adult survivorship >0.80 very few parameter combinations resulted in population growth rates >0 (although some of these were as large as 10%). A logical next step to our analysis would be a direct comparison between MPM predictions for $\lambda_{\max}$ with the output of the Niel and Lebreton (2005) approach used here. Collectively these studies are consistent with our estimates of *R*_max_ and show that loggerheads are expected to have relatively slow recovery rates even under the best of demographic conditions.

Because we used an indirect method to estimate *R*_max_, there is uncertainty in the approach with one known bias resulting in possible overestimation. The demographic invariant method (DIM) used to estimate $\lambda_{\max}$ assumes that the input life‐history parameters were “observed” in demographically optimal conditions. Bias can result if there was any anthropogenic mortality or sampling error left unaccounted for in the variability of the life‐history parameter estimates. Dillingham et al. (2016) showed that the DIM approach was more sensitive to adult survival than age‐at‐first‐reproduction in calculating $\lambda_{\max}$, and we used estimates for adult survival rates that attempted to adjust for fisheries‐related mortality (bycatch). Even so, if the assumed adult survival estimates were in fact lower than what could be achieved naturally under optimal conditions, the maximum population growth rate presented here would be overestimated. It may be best, then, to view the distribution of ${\hat{R}}_{\max}$ as an upper bound for maximum population growth in a management context or as a prior distribution in estimating trends from empirical nesting beach or aerial survey data.

A comparative analysis of annual maximum population growth rates across loggerhead populations would be an avenue for future research. Other loggerhead populations have demonstrated observed annual trends in nesting that exceeded our median estimated biological maximum for the NWA population (Baldwin, Hughes, & Prince, 2003; Marcovaldi & Chaloupka, 2007). Although, as we stated previously, relating trends in nesting to the annual maximum population growth rate can be tenuous. Differences in intrinsic ecological factors or anthropogenic influences across populations could affect observed trends at nesting beach sites, including deviations from the stable age distribution. Furthermore, our estimated distribution of the annual maximum population growth rate is a result of the assumed or developed distribution of adult survival and age‐at‐first‐reproduction. It is possible that under some conditions and more refined determination of adult survival and age‐at‐first‐reproduction the NWA loggerhead population could achieve a population growth rate greater than what was estimated here.

## CONFLICT OF INTEREST

None declared.

## AUTHORS’ CONTRIBUTIONS

JMH conceived, designed, conducted the analysis, and wrote the first draft of the manuscript. HLH, PMR, and KAR contributed to the interpretation of the results and edited drafts of the manuscript.

## Data Availability

No data were collected during this analysis. Relevant data were either provided in the manuscript or were sourced from publicly accessible repositories or manuscripts.
